# Association between inflammation- and nutrition-related indicators and mortality in patients with heart failure: a cohort study

**DOI:** 10.3389/fnut.2025.1617069

**Published:** 2025-10-28

**Authors:** Songtao Liu, Ting Fu, Tianhua Deng, Xinyong Cai, Yuliang Zhan, Hongmin Zhu

**Affiliations:** ^1^Department of Cardiology, Jiangxi Provincial People’s Hospital, The First Affiliated Hospital of Nanchang Medical College, Nanchang, China; ^2^Department of Cardiac Electrophysiology, Jiangxi Provincial People’s Hospital, The First Affiliated Hospital of Nanchang Medical College, Nanchang, China

**Keywords:** heart failure, inflammation- and nutrition-related indicators, mortality, nutritional status, inflammation

## Abstract

**Objective:**

Inflammation and malnutrition are critical in heart failure (HF) progression. This study evaluated the prognostic value of inflammation- and nutrition-related indicators for mortality in HF.

**Methods:**

Retrospective analysis of 1999–2018 NHANES data (101,316 participants, 1,500 HF patients) assessed indicators including advanced lung cancer inflammation index (ALI), monocyte-to-albumin ratio (MAR), neutrophil-to-albumin ratio (NAR), red cell distribution width-to-albumin ratio (RAR), prognostic nutritional index (PNI), geriatric nutritional risk index (GNRI), hemoglobin-albumin-lymphocyte-platelet (HALP) score and controlling nutritional status (CONUT) score. Associations with all-cause and cardiovascular mortality were analyzed via Kaplan–Meier curves, Cox regression, restricted cubic spline, time-dependent ROC, and random survival forest (RSF).

**Results:**

A total of 1,500 HF patients were included in the final analysis. Kaplan–Meier analysis demonstrated that elevated MAR, NAR, RAR, and CONUT scores were linked to higher mortality, whereas elevated ALI, PNI, GNRI, and HALP scores were associated with lower mortality in HF patients. After false discovery rate (FDR) correction, the majority of indicators (including ALI, RAR) remained significantly associated with mortality in multivariable Cox models. Time-dependent ROC analysis demonstrated that RAR exhibited the strongest predictive ability for 1-year all-cause mortality (AUC = 0.768, 95% CI: 0.718–0.819) and cardiovascular mortality (AUC = 0.788, 95% CI: 0.725–0.851). In contrast, ALI showed the best predictive performance for mortality at 3 years (all-cause: AUC = 0.690, 95% CI: 0.654–0.726; cardiovascular: AUC = 0.705, 95% CI: 0.655–0.756), 5 years (all-cause: AUC = 0.679, 95% CI: 0.647–0.711; cardiovascular: AUC = 0.677, 95% CI: 0.633–0.721), and 10 years (all-cause: AUC = 0.691, 95% CI: 0.657–0.725; cardiovascular: AUC = 0.699, 95% CI: 0.656–0.742). These findings were consistent with the C-index results. RSF analysis, validated by an internal hold-out test, consistently identified ALI as a leading predictor of mortality risk.

**Conclusion:**

Compared with other inflammation- and nutrition-related indicators, RAR and ALI may provide superior predictive value for short-term and long-term mortality risk, respectively, in HF patients.

## Introduction

Heart failure (HF) has become a significant global public health challenge, with its prevalence steadily increasing, largely driven by the accelerating aging of the population worldwide (1). HF patients have a poor prognosis, with an annual mortality rate reaching up to 33%, and studies encompassing all adult age groups report an overall mortality rate of 24% (2). Moreover, the frequent hospitalizations associated with HF not only impose a substantial burden on healthcare resources but also have a profound negative impact on patients’ quality of life (3).

Malnutrition is highly prevalent among HF patients, with an estimated incidence ranging from 15 to 90%, depending on the assessment method used (4). Several mechanisms contribute to malnutrition in HF, including activation of the renin-angiotensin-aldosterone system (RAAS), involvement of inflammatory mediators, reduced cardiac output, and appetite loss due to dyspnea or intestinal edema (5). In addition, chronic illness, prolonged physical inactivity, and extended bed rest can lead to muscle wasting, further exacerbating malnutrition (6). Studies indicate that HF patients with malnutrition have nearly twice the all-cause mortality rate compared to their well-nourished counterparts, highlighting the critical role of malnutrition in increasing mortality risk (7).

Systemic inflammation is widely recognized as a key pathophysiological feature of both acute and chronic HF, playing a pivotal role in disease progression, exacerbation, and the development of complications (8). Recent studies have highlighted a significant link between inflammatory biomarkers and the prognosis of HF (9). As a result, indices that evaluate both inflammation and nutritional status in a comprehensive manner hold promise as more effective prognostic tools.

There is growing interest in inflammation- and nutrition-related indicators. The Advanced Lung Cancer Inflammation Index (ALI) serves as a composite biomarker that assesses inflammation and nutritional status. Originally designed to evaluate the degree of systemic inflammation in patients with metastatic non-small cell lung cancer (NSCLC) (10), ALI is computed using the formula: body mass index (BMI) multiplied by serum albumin (Alb) divided by the neutrophil to lymphocyte ratio (NLR). Due to its effectiveness in reflecting the body’s inflammatory and nutritional states, ALI has been applied in various disease populations similarly impacted by nutrition and inflammation, including cancer (11), rheumatoid arthritis (12), and diabetes (13). The red cell distribution width-to-albumin ratio (RAR = RDW/albumin) is a novel composite biomarker that reflects both inflammatory and nutritional status. In recent years, multiple studies have demonstrated that RAR is closely associated with mortality risk in a range of critical conditions, including acute myocardial infarction (14), acute respiratory failure (15), and sepsis (16). It has shown strong prognostic utility in these clinical settings.

Additionally, there has been extensive research on other inflammation- and nutrition-related indices, including the monocyte-to-albumin ratio (MAR), neutrophil-to-albumin ratio (NAR), prognostic nutritional index (PNI), geriatric nutritional risk index (GNRI), hemoglobin-albumin-lymphocyte-platelet (HALP) score, and controlling nutritional status (CONUT) score (17, 18). Different inflammation- and nutrition-related biomarkers may reflect distinct pathophysiological aspects of HF, and their predictive value and clinical utility may vary. However, comprehensive evidence on the association between these derived indicators and HF-related mortality is currently lacking, and no studies have systematically compared the prognostic value of these indices for predicting all-cause and cardiovascular mortality in HF patients. Furthermore, while some studies suggest that inflammation- and nutrition-related indicators may be valuable for survival prediction in HF, the optimal prognostic marker remains uncertain.

Therefore, this study utilized data from the nationally representative National Health and Nutrition Examination Survey (NHANES) cohort to investigate the association between inflammation- and nutrition-related indicators and HF prognosis. By systematically evaluating and comparing the prognostic value of these indices, this study aims to clarify their clinical significance and provide more comprehensive evidence to support risk stratification and precision management in HF patients.

## Materials and methods

### Study population

**Figure 1:**
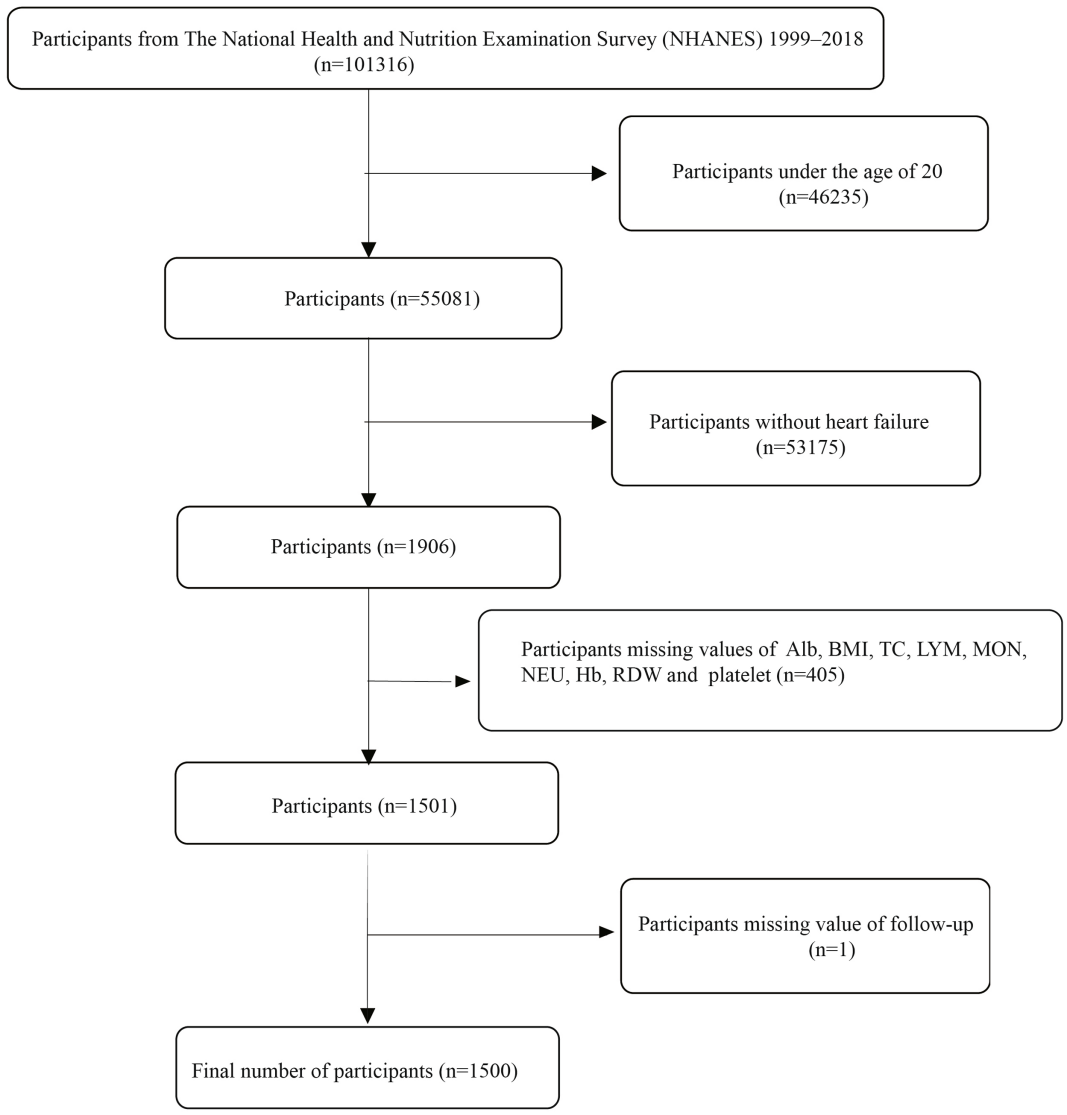
The flow chart.

This study utilized publicly available data from the National Health and Nutrition Examination Survey (NHANES) spanning 1999–2018. The NHANES study received approval from the Ethics Review Board of the National Center for Health Statistics (NCHS), and all participants provided written informed consent before enrollment. The NHANES 1999–2018 cycles included a total of 101,316 participants. The inclusion criteria for this study were defined as follows: (i) self-reported heart failure, defined as an affirmative response to the question, “Has a doctor or other health professional ever told you that you have congestive heart failure?”; (ii) age ≥20 years; and (iii) availability of relevant data on complete blood count, albumin, total cholesterol, and body measurements. Exclusion criteria included: (i) age <20 years, (ii) missing data for inflammation/nutrition-related indicators, and (iii) missing follow-up data. Based on the above criteria, a total of 1,500 HF patients were finally included in this study. The process of participant selection is illustrated in Figure 1. All data used in this research are publicly available on the NHANES website: https://www.cdc.gov/nchs/nhanes/.

### Assessment of inflammation and nutritional indicators

Inflammation and nutritional indicators were assessed using multiple hematological and biochemical parameters, including neutrophils, lymphocytes, monocytes, hemoglobin, and red cell distribution width (RDW) from complete blood count, as well as serum albumin, total cholesterol, and body mass index (BMI). Blood samples were analyzed at NHANES mobile examination centers (MECs) using the Beckman Coulter DxH 800 analyzer for hematologic parameters, while serum albumin and total cholesterol levels were measured using the Roche Cobas 6000 analyzer (c501 module). Height, weight, and BMI were measured at the MECs.

Based on these individual inflammation and nutrition markers, we calculated composite inflammation/nutrition-related indicators, including the advanced lung cancer inflammation index (ALI), monocyte-to-albumin ratio (MAR), neutrophil-to-albumin ratio (NAR), red cell distribution width-to-albumin ratio (RAR), prognostic nutritional index (PNI), geriatric nutritional risk index (GNRI), hemoglobin, albumin, lymphocyte, and platelet (HALP) score, and the controlling nutritional status (CONUT) score. Participants were divided into quartiles (Q1, Q2, Q3, and Q4) for each composite index to facilitate further analysis. Detailed formulas for each inflammation/nutrition index are provided in the Supplementary Table S1.

### Primary outcome

The primary outcomes of this study were all-cause mortality and cardiovascular mortality during follow-up. The mortality status was ascertained using data from the National Death Index (NDI) through December 31, 2019. The mortality records are available for public access at the following link: https://www.cdc.gov/nchs/data-linkage/mortality.htm. The cause of death was classified based on the International Classification of Diseases, 10th Edition (ICD-10).

### Covariates definitions

To minimize confounding bias, several potential covariates were included in the analysis, including (i) sociodemographic characteristics: age, sex, race, and education level; (ii) lifestyle factors: smoking status and alcohol consumption; (iii) medical history: hypertension, diabetes, coronary artery disease, stroke, and cancer; and (iv) laboratory parameters: glycated hemoglobin (HbA1c), estimated glomerular filtration rate (eGFR), alanine aminotransferase (ALT), aspartate aminotransferase (AST). Detailed definitions of these covariates can be found at the following link: https://www.cdc.gov/nchs/nhanes/.

### Statistical analysis

All statistical analyses were performed using SPSS version 25.0 (IBM, Chicago, IL, United States) and R software version 4.3.0. Continuous variables with a normal distribution are expressed as means ± standard errors (SE), while those without a normal distribution are presented as medians (interquartile ranges). Categorical variables are shown as frequencies (percentages). A two-tailed *p*-value <0.05 was considered statistically significant.

To assess the long-term relationship between inflammation/nutrition-related indicators and mortality in HF patients, Kaplan–Meier survival curves were constructed, and differences between groups were evaluated using the log-rank test. Cox proportional hazards regression models were employed to examine the relationship between inflammation/nutrition-related indicators and both all-cause and cardiovascular mortality in HF patients. Three models with different levels of covariate adjustments were constructed: (i) basic model: unadjusted; (ii) Model 1: adjusted for age, sex, race, education level, smoking status, and alcohol consumption; and (iii) Model 2: further adjusted for hypertension, diabetes, coronary artery disease, stroke, cancer history, HbA1c, eGFR, alanine aminotransferase (ALT), and aspartate aminotransferase (AST). These models accounted for potential confounders to enhance the robustness and reliability of the results. The Generalized Variance Inflation Factor (GVIF) was calculated to assess multicollinearity among covariates in the fully adjusted models, with all GVIF^(1/(2*Df))^ values below 5 indicating the absence of severe multicollinearity. To account for multiple testing across the eight indicators and two outcomes, false discovery rate (FDR) correction was applied.

To investigate potential nonlinear relationships between inflammation/nutrition-related indicators and mortality risk, restricted cubic spline (RCS) regression models with four knots (located at the 25th, 50th, 75th, and 90th percentiles) were applied. To assess the prognostic value of each index at various follow-up time points (1-year, 3-year, 5-year, and 10-year), time-dependent receiver operating characteristic (ROC) curve analysis was conducted, and the corresponding area under the curve (AUC) values and concordance index (C-index) were calculated.

Furthermore, a random survival forest (RSF) analysis was performed to provide a data-driven assessment of variable importance for mortality prediction. To mitigate the risk of overfitting and validate the stability of the findings, the dataset was randomly split into a training set (70%) and an independent internal validation set (30%); the model was built on the training set and its variable importance ranking was evaluated on the hold-out validation set.

## Results

### Baseline characteristics

The baseline characteristics of the study population are summarized in Table 1. A total of 1,500 heart failure patients were included, with a mean age of 67.59 ± 0.33 years. Of the total participants, 56.20% were male, while 43.80% were female. In terms of racial distribution, 820 participants (54.67%) were non-Hispanic White. Additionally, 61.11% of the participants were smokers. The prevalence of hypertension and diabetes was 81.87 and 45.87%, respectively.

**Table 1:** Baseline characteristics of patients with heart failure in NHANES 1999–2018.

Character	Total
Age	70 (60, 79)
Gender, *n* (%)	
Female	657 (43.80)
Male	843 (56.20)
Race, *n* (%)	
Mexican American	162 (10.80)
Non-Hispanic White	820 (54.67)
Non-Hispanic Black	343 (22.87)
Others	175 (11.67)
Education level, *n* (%)	
Below high school	591 (39.48)
High school	367 (24.52)
Above high school	539 (36.01)
Smoke, *n* (%)	
No	583 (38.89)
Yes	916 (61.11)
Drink, *n* (%)	
No	214 (15.20)
Yes	1,194 (84.80)
Hypertension, *n* (%)	
No	272 (18.13)
Yes	1,228 (81.87)
Diabetes, *n* (%)	
No	812 (54.13)
Yes	688 (45.87)
Coronary artery disease, *n* (%)	
No	844 (58.21)
Yes	606 (41.79)
Stroke, *n* (%)	
No	1,194 (79.81)
Yes	302 (20.19)
Cancer, *n* (%)	
No	1,184 (79.20)
Yes	311 (20.80)
HbA1c, %	5.9 (5.5, 6.6)
eGFR, mL/min/1.73 m^2^	66.61 (47.93, 86.65)
ALT, mmol/L	19 (15, 25)
AST, mmol/L	23 (19, 28)
Inflammation and nutritional indicators	
Lymphocyte, 10^3^/μL	1.8 (1.4, 2.4)
Monocyte, 10^3^/μL	0.6 (0.5, 0.7)
Neutrophil, 10^3^/μL	4.4 (3.5, 5.6)
Hemoglobin, g/dL	13.6 (12.5, 14.7)
RDW, %	13.7 (12.9, 14.8)
Platelet, 10^3^/μL	218.5 (179, 267)
BMI, kg/m^2^	30.10 (25.98, 35.5)
Total cholesterol, mmol/L	4.55 (3.75, 5.38)
Serum albumin, g/L	41 (38, 43)
Inflammation/nutrition-based indicators	
ALI	52.71 (34.72, 75.52)
MAR	0.015 (0.012, 0.018)
NAR	0.11 (0.08, 0.14)
RAR	0.338 (0.308, 0.376)
PNI	50.25 (47.00, 54.00)
GNRI	118.05 (109.42, 128.14)
HALP score	46.11 (32.50, 64.00)
CONUT score	1 (0, 2)

HbA1c, glycosylated hemoglobin, type A1C; eGFR, estimated glomerular filtration rate; AST, aspartate aminotransferase; ALT, alanine aminotransferase; RDW, red cell distribution width; BMI, body mass index; ALI, advanced lung cancer inflammation index; MAR, monocyte- to- albumin ratio; NAR, neutrophil- to- albumin ratio; RAR, red cell distribution width-albumin ratio; PNI, prognostic nutritional index; GNRI, geriatric nutrition risk index; HALP, hemoglobin, albumin, lymphocyte, and platelet; CONUT, controlling nutritional status.

### Relationship between inflammation/nutrition-related indicators and mortality

**Figure 2:**
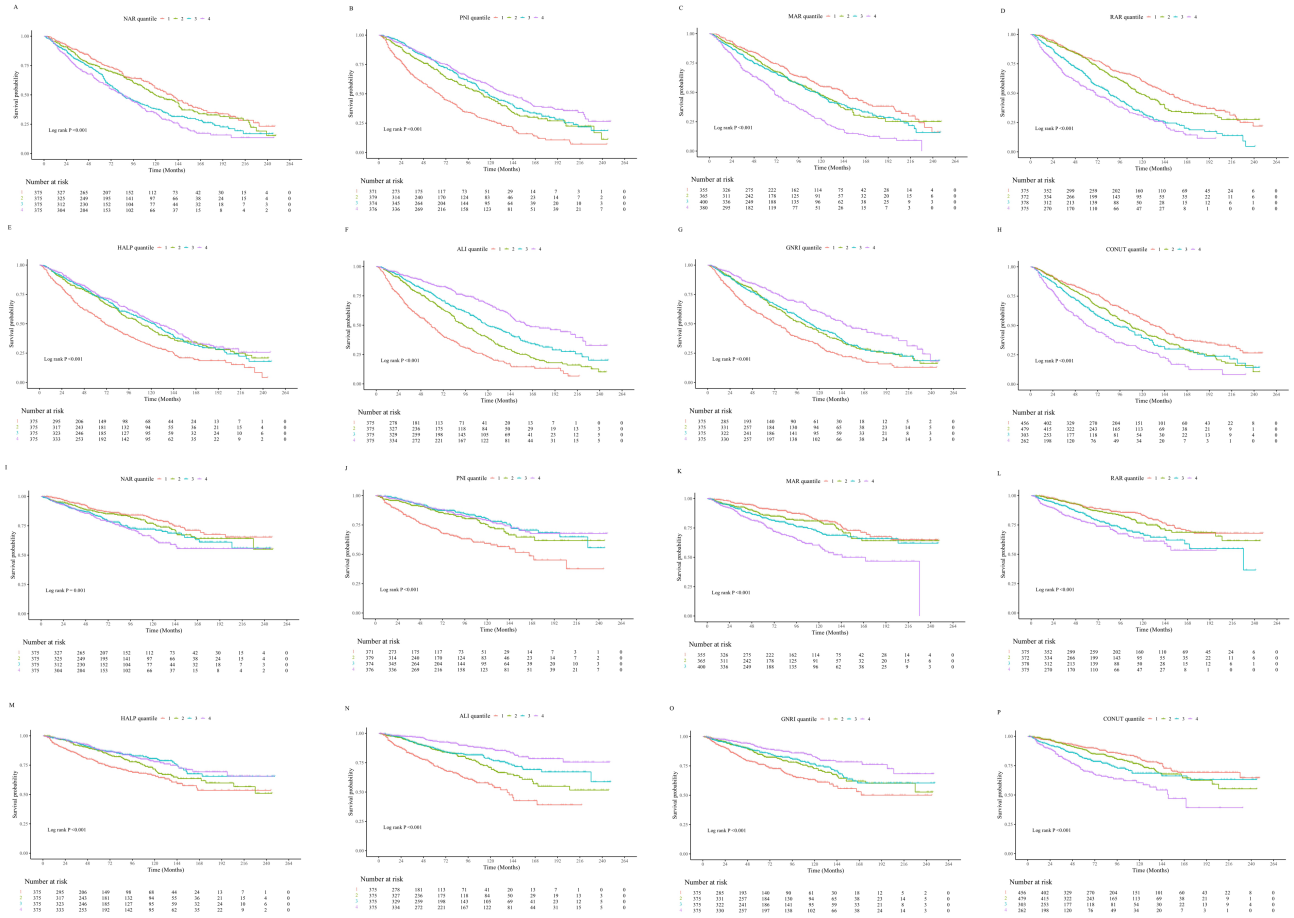
**(A–H)** Kaplan–Meier survival curves illustrating the association between inflammation- and nutrition-related indicators and all-cause mortality in patients with heart failure. **(I–P)** Kaplan–Meier survival curves illustrating the association between inflammation- and nutrition-related indicators and cardiovascular disease mortality in patients with heart failure.

The mean follow-up duration was 79.48 ± 1.46 months. Over the follow-up period, a total of 786 patients (52.4%) experienced all-cause mortality, of whom 305 (20.33%) died from cardiovascular causes. Kaplan–Meier survival analysis demonstrated that as the quartiles of RAR, NAR, MAR, and CONUT score increased, the cumulative incidence of all-cause and cardiovascular mortality significantly rose (log-rank test, all *p* < 0.001). Conversely, higher quartiles of ALI, PNI, GNRI, and HALP score were significantly associated with lower cumulative all-cause and cardiovascular mortality rates (log-rank test, all *p* < 0.001) (Figure 2).

As shown in Table 2, the results of the Cox regression analysis demonstrated that all inflammation/nutrition-based indicators were significantly associated with all-cause mortality in both the crude model and the minimally adjusted model (Model 1). In the fully adjusted model (Model 2), the GVIF^(1/(2*Df))^ values for all covariates were below 5, indicating no severe multicollinearity (see Supplementary Table S2). After false discovery rate (FDR) correction for multiple testing, all indicators remained statistically significant for all-cause mortality. For cardiovascular mortality, the associations for NAR and HALP were no longer significant after FDR correction (*q* > 0.05), while the other six indicators remained robust predictors.

**Table 2:** Univariate and multivariate Cox analysis of the association between all-cause and CVD mortality and inflammation/nutrition-based indicators in heart failure patients.

Indicators	All-cause mortality			CVD mortality		
	Crude	Model 1	Model 2	Crude	Model 1	Model 2
	HR (95% CI)	HR (95% CI)	HR (95% CI)	HR (95% CI)	HR (95% CI)	HR (95% CI)
ALI						
Quantile 1	Reference	Reference	Reference	Reference	Reference	Reference
Quantile 2	0.59 (0.49–0.70)	0.67 (0.56–0.81)	0.70 (0.57–0.85)	0.52 (0.39–0.69)	0.59 (0.43–0.80)	0.59 (0.43–0.81)
Quantile 3	0.43 (0.35–0.52)	0.57 (0.46–0.70)	0.60 (0.48–0.74)	0.40 (0.30–0.55)	0.57 (0.42–0.79)	0.58 (0.41–0.81)
Quantile 4	0.27 (0.21–0.33)	0.45 (0.35–0.56)	0.49 (0.39–0.63)	0.23 (0.16–0.33)	0.39 (0.26–0.57)	0.44 (0.29–0.66)
*p* for trend	<0.001	<0.001	<0.001	<0.001	<0.001	<0.001
FDR *q* value	<0.001	<0.001	<0.001	<0.001	<0.001	<0.001
MAR						
Quantile 1	Reference	Reference	Reference	Reference	Reference	Reference
Quantile 2	1.25 (1.01–1.54)	1.17 (0.94–1.47)	1.13 (0.90–1.42)	1.28 (0.90–1.83)	1.22 (0.83–1.78)	1.13 (0.77–1.66)
Quantile 3	1.32 (1.08–1.62)	1.22 (0.98–1.52)	1.11 (0.89–1.39)	1.56 (1.12–2.18)	1.53 (1.08–2.18)	1.35 (0.94–1.95)
Quantile 4	2.29 (1.88–2.80)	1.90 (1.54–2.36)	1.65 (1.32–2.06)	2.57 (1.85–3.56)	2.28 (1.61–3.23)	1.95 (1.35–2.80)
*p* for trend	<0.001	<0.001	<0.001	<0.001	<0.001	<0.001
FDR *q* value	<0.001	<0.001	<0.001	<0.001	<0.001	<0.001
NAR						
Quantile 1	Reference	Reference	Reference	Reference	Reference	Reference
Quantile 2	1.15 (0.93–1.42)	0.94 (0.75–1.17)	0.91 (0.73–1.14)	1.22 (0.87–1.71)	1.03 (0.72–1.47)	0.97 (0.68–1.40)
Quantile 3	1.55 (1.26–1.89)	1.24 (1.00–1.55)	1.17 (0.93–1.46)	1.54 (1.11–2.14)	1.24 (0.87–1.77)	1.12 (0.78–1.62)
Quantile 4	1.84 (1.51–2.24)	1.65 (1.33–2.04)	1.40 (1.12–1.76)	1.82 (1.31–2.51)	1.73 (1.22–2.44)	1.41 (0.98–2.03)
*p* for trend	<0.001	<0.001	<0.001	<0.001	<0.001	0.036
FDR *q* value	<0.001	<0.001	0.002	<0.001	0.004	0.187
RAR						
Quantile 1	Reference	Reference	Reference	Reference	Reference	Reference
Quantile 2	1.21 (0.99–1.49)	1.25 (1.01–1.55)	1.18 (0.94–1.47)	1.17 (0.84–1.64)	1.21 (0.86–1.71)	1.21 (0.84–1.73)
Quantile 3	1.96 (1.60–2.40)	2.14 (1.73–2.64)	1.90 (1.53–2.37)	1.84 (1.33–2.54)	2.06 (1.47–2.89)	1.86 (1.30–2.66)
Quantile 4	2.51 (2.06–3.06)	2.90 (2.34–3.60)	2.43 (1.94–3.06)	2.42 (1.76–3.32)	2.78 (1.97–3.92)	2.40 (1.66–3.47)
*p* for trend	<0.001	<0.001	<0.001	<0.001	<0.001	<0.001
FDR *q* value	<0.001	<0.001	<0.001	<0.001	<0.001	<0.001
PNI						
Quantile 1	Reference	Reference	Reference	Reference	Reference	Reference
Quantile 2	0.56 (0.46–0.68)	0.53 (0.43–0.65)	0.59 (0.48–0.72)	0.48 (0.35–0.65)	0.46 (0.33–0.63)	0.51 (0.36–0.71)
Quantile 3	0.48 (0.39–0.58)	0.46 (0.38–0.57)	0.51 (0.41–0.64)	0.39 (0.28–0.53)	0.39 (0.28–0.54)	0.43 (0.30–0.61)
Quantile 4	0.40 (0.33–0.49)	0.48 (0.39–0.60)	0.58 (0.47–0.72)	0.39 (0.29–0.54)	0.48 (0.34–0.66)	0.56 (0.40–0.79)
*p* for trend	<0.001	<0.001	<0.001	<0.001	<0.001	<0.001
FDR *q* value	<0.001	<0.001	<0.001	<0.001	<0.001	0.002
GNRI						
Quantile 1	Reference	Reference	Reference	Reference	Reference	Reference
Quantile 2	0.66 (0.55–0.80)	0.67 (0.55–0.81)	0.65 (0.53–0.79)	0.64 (0.48–0.86)	0.63 (0.46–0.86)	0.59 (0.43–0.82)
Quantile 3	0.64 (0.53–0.77)	0.70 (0.57–0.85)	0.67 (0.54–0.82)	0.58 (0.43–0.79)	0.63 (0.46–0.87)	0.57 (0.41–0.80)
Quantile 4	0.43 (0.35–0.54)	0.65 (0.52–0.81)	0.59 (0.47–0.76)	0.38 (0.27–0.54)	0.57 (0.40–0.83)	0.48 (0.32–0.71)
*p* for trend	<0.001	<0.001	<0.001	<0.001	0.002	<0.001
FDR *q* value	<0.001	0.002	<0.001	<0.001	0.012	0.001
HALP score						
Quantile 1	Reference	Reference	Reference	Reference	Reference	Reference
Quantile 2	0.63 (0.52–0.76)	0.68 (0.56–0.83)	0.70 (0.57–0.86)	0.69 (0.51–0.92)	0.78 (0.57–1.06)	0.78 (0.56–1.07)
Quantile 3	0.60 (0.49–0.72)	0.68 (0.55–0.83)	0.72 (0.58–0.89)	0.51 (0.37–0.71)	0.59 (0.42–0.83)	0.59 (0.41–0.84)
Quantile 4	0.54 (0.44–0.66)	0.66 (0.53–0.81)	0.72 (0.57–0.89)	0.52 (0.38–0.71)	0.63 (0.44–0.88)	0.69 (0.48–0.98)
*p* for trend	<0.001	<0.001	0.004	<0.001	0.002	0.013
FDR *q* value	<0.001	<0.001	0.026	<0.001	0.010	0.08
CONUT score						
Quantile 1	Reference	Reference	Reference	Reference	Reference	Reference
Quantile 2	1.32 (1.10–1.59)	1.11 (0.91–1.34)	1.00 (0.82–1.22)	1.27 (0.94–1.72)	1.09 (0.80–1.49)	0.99 (0.71–1.37)
Quantile 3	1.54 (1.25–1.89)	1.18 (0.94–1.47)	1.04 (0.82–1.30)	1.65 (1.18–2.30)	1.30 (0.91–1.85)	1.19 (0.83–1.72)
Quantile 4	2.34 (1.90–2.87)	1.71 (1.38–2.13)	1.48 (1.17–1.86)	2.72 (1.98–3.76)	1.93 (1.37–2.72)	1.70 (1.18–2.44)
*p* for trend	<0.001	<0.001	0.002	<0.001	<0.001	0.003
FDR *q* value	<0.001	<0.001	0.013	<0.001	<0.001	0.017

Crude model: No adjustments were made.

Model 1: Adjusted for age, sex, race, education level, smoking status, and alcohol consumption.

Model 2: Further adjusted for hypertension, diabetes, coronary artery disease, stroke, cancer, glycated hemoglobin (HbA1c, %), estimated glomerular filtration rate (mL/min/1.73 m^2^), aspartate aminotransferase (U/L), and alanine aminotransferase (U/L) based on Model 1.

HR, hazard ratios; 95% CI, 95% confidence interval; ALI, advanced lung cancer inflammation index; MAR, monocyte- to- albumin ratio; NAR, neutrophil- to- albumin ratio; RAR, red cell distribution width-albumin ratio; PNI, prognostic nutritional index; GNRI, geriatric nutrition risk index; HALP, hemoglobin, albumin, lymphocyte, and platelet; CONUT, controlling nutritional status.

### Threshold effect analysis of inflammation/nutrition-based indicators and their impact on mortality risk

**Figure 3:**
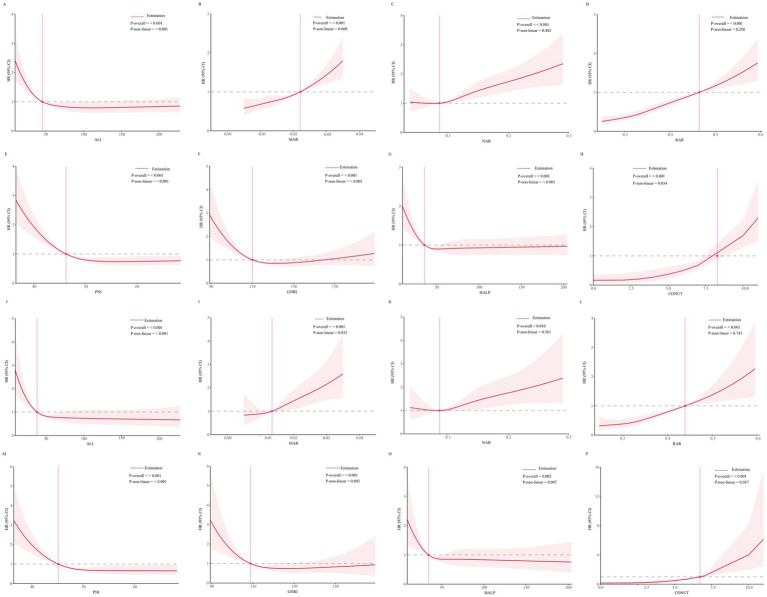
**(A–H)** Threshold effect analysis of inflammation/nutrition-based indicators on all-cause mortality. **(I–P)** Threshold effect analysis of inflammation/nutrition-based indicators on cardiovascular disease mortality.

RCS analysis revealed a linear relationship (*p* > 0.05) between MAR, NAR, RAR, and CONUT score with all-cause mortality risk, whereas ALI, PNI, GNRI and HALP score exhibited a nonlinear relationship with all-cause mortality risk (*p* < 0.05). Similarly, the associations between MAR, NAR, RAR, and CONUT score with cardiovascular mortality risk were linear (*p* > 0.05), while those of ALI, PNI, GNRI and HALP score were nonlinear (*p* < 0.05). Details are presented in Figure 3.

### Prediction of mortality by inflammation/nutrition-based indicators

**Figure 4:**
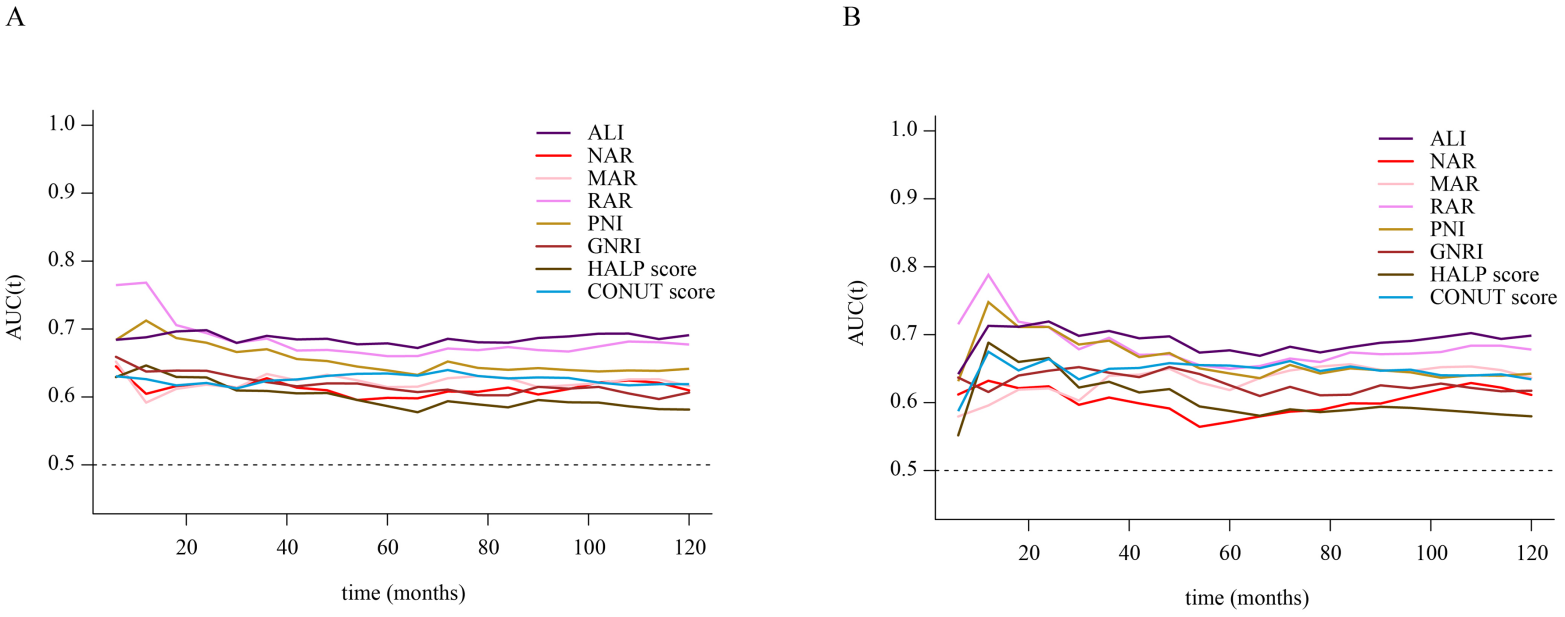
**(A)** Time-dependent ROC curves of inflammation/nutrition-based indicators for predicting all-cause mortality in patients with heart failure. **(B)** Time-dependent ROC curves of inflammation/nutrition-based indicators for predicting cardiovascular disease mortality in patients with heart failure.

Time-dependent ROC curve analysis was conducted to evaluate the prognostic performance of inflammation/nutrition-related indicators in predicting 1-year, 3-year, 5-year, and 10-year all-cause and cardiovascular mortality in HF patients (Figure 4). The results (Tables 3, 4) showed that, compared to other inflammation/nutrition-based indicators, RAR exhibited the strongest predictive ability for 1-year all-cause and cardiovascular mortality, with AUCs of 0.768 (95% CI: 0.718, 0.819) and 0.788 (95% CI: 0.725, 0.851), respectively. However, ALI demonstrated the highest predictive performance for 3-year, 5-year, and 10-year all-cause mortality, with AUCs of 0.690 (95% CI: 0.654, 0.726), 0.679 (95% CI: 0.647, 0.711), and 0.691 (95% CI: 0.657, 0.725), respectively. Similarly, ALI exhibited the best predictive value for 3-year, 5-year, and 10-year cardiovascular mortality, with AUCs of 0.705 (95% CI: 0.655, 0.756), 0.677 (95% CI: 0.633, 0.721), and 0.699 (95% CI: 0.656, 0.742), respectively. The overall discriminative ability assessed by the concordance index (C-index) yielded consistent results with the time-dependent AUC analyses (see Supplementary Tables S3, S4).

**Table 3:** Time-dependent ROC analysis: AUC values of inflammation/nutrition-based indicators for predicting all-cause mortality in heart failure patients.

Indicators	AUC (95% CI)			
	1-year	3-year	5-years	10-years
ALI	0.688 (0.622, 0.754)	**0.690 (0.654, 0.726)**	**0.679 (0.647, 0.711)**	**0.691 (0.657, 0.725)**
MAR	0.592 (0.528, 0.655)	0.634 (0.596, 0.672)	0.615 (0.581, 0.648)	0.616 (0.581, 0.652)
NAR	0.605 (0.543, 0.666)	0.627 (0.590, 0.665)	0.599 (0.565, 0.632)	0.610 (0.573, 0.646)
RAR	**0.768 (0.718, 0.819)**	0.686 (0.649, 0.722)	0.660 (0.627, 0.693)	0.677 (0.643, 0.712)
PNI	0.712 (0.646, 0.779)	0.670 (0.632, 0.709)	0.639 (0.605, 0.673)	0.641 (0.607, 0.676)
GNRI	0.637 (0.567, 0.708)	0.622 (0.584, 0.660)	0.612 (0.579, 0.646)	0.606 (0.570, 0.643)
HALP score	0.646 (0.577, 0.716)	0.609 (0.570, 0.648)	0.587 (0.52, 0.621)	0.581 (0.545, 0.618)
CONUT score	0.626 (0.557, 0.695)	0.624 (0.586, 0.662)	0.635 (0.602, 0.667)	0.619 (0.584, 0.653)

AUC, area under the curve; 95% CI, 95% confidence interval; ALI, advanced lung cancer inflammation index; MAR, monocyte- to- albumin ratio; NAR, neutrophil- to- albumin ratio; RAR, red cell distribution width-albumin ratio; PNI, prognostic nutritional index; GNRI, geriatric nutrition risk index; HALP, hemoglobin, albumin, lymphocyte, and platelet; CONUT, controlling nutritional status. Bold values represent the highest AUC values across different predictors at each specific time point.

**Table 4:** Time-dependent ROC analysis: AUC values of inflammation/nutrition-based indicators for predicting CVD mortality in heart failure patients.

Indicators	AUC (95% CI)			
	1-years	3-years	5-years	10-years
ALI	0.713 (0.625, 0.801)	**0.705 (0.655, 0.756)**	**0.677 (0.633, 0.721)**	**0.699 (0.656, 0.742)**
MAR	0.596 (0.515, 0.677)	0.640 (0.588, 0.691)	0.618 (0.574, 0.663)	0.636 (0.589, 0.683)
NAR	0.632 (0.558, 0.707)	0.607 (0.556, 0.660)	0.572 (0.526, 0.617)	0.611 (0.564, 0.658)
RAR	**0.788 (0.725, 0.851)**	0.695 (0.643, 0.748)	0.650 (0.604, 0.695)	0.678 (0.633, 0.723)
PNI	0.748 (0.666, 0.830)	0.691 (0.636, 0.747)	0.643 (0.596, 0.690)	0.642 (0.597, 0.688)
GNRI	0.616 (0.522, 0.709)	0.644 (0.590, 0.698)	0.626 (0.580, 0.671)	0.618 (0.572, 0.663)
HALP score	0.688 (0.594, 0.783)	0.631 (0.575, 0.686)	0.588 (0.540, 0.636)	0.580 (0.532, 0.628)
CONUT score	0.675 (0.584, 0.765)	0.650 (0.595, 0.704)	0.655 (0.610, 0.699)	0.634 (0.590, 0.679)

AUC, area under the curve; 95% CI, 95% confidence interval; ALI, advanced lung cancer inflammation index; MAR, monocyte- to- albumin ratio; NAR, neutrophil- to- albumin ratio; RAR, red cell distribution width-albumin ratio; PNI, prognostic nutritional index; GNRI, geriatric nutrition risk index; HALP, hemoglobin, albumin, lymphocyte, and platelet; CONUT, controlling nutritional status. Bold values represent the highest AUC values across different predictors at each specific time point.

### Random survival forest analysis

An RSF model was developed to assess predictor importance. To validate its robustness, the analysis was performed using a 70/30 training/validation split. ALI consistently demonstrated the highest variable importance for mortality prediction in both the training and independent validation sets. The relative ranking of all indicators is shown in Supplementary Figure S1.

### Stratified analysis and subgroup analysis

To further explore the prognostic value of inflammation/nutrition-based markers in different populations, stratified analyses were performed based on age, sex, BMI, diabetes, and hypertension status. The results showed that ALI consistently exhibited a strong predictive ability for all-cause and cardiovascular mortality across all subgroups, further validating its clinical utility. Detailed data are presented in Supplementary Tables S5–S9.

**Figure 5:**
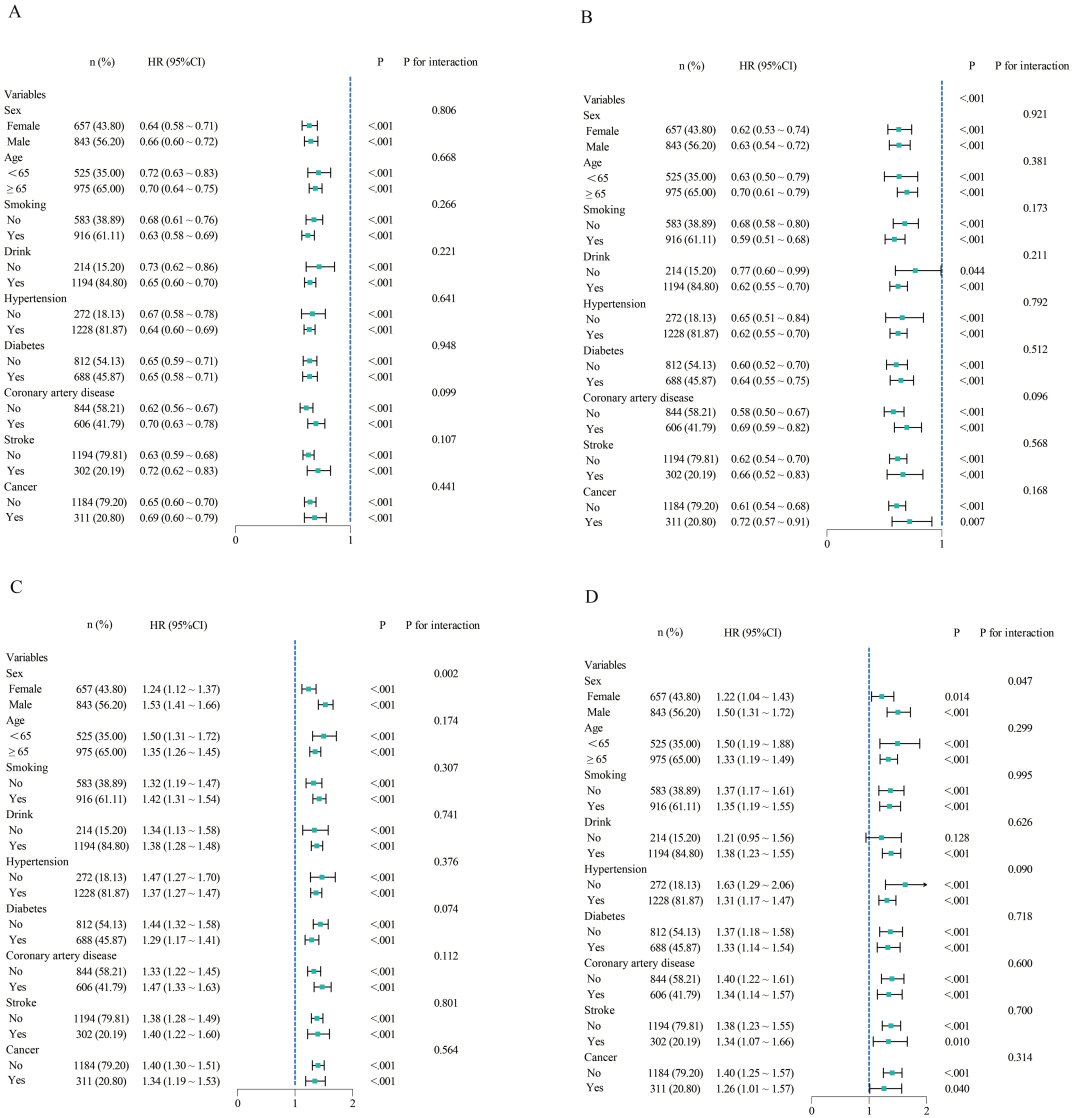
Subgroup analysis of ALI and RAR for all-cause and CVD mortality. **(A,B)** Subgroup analysis of ALI for all-cause and cardiovascular mortality. **(C,D)** Subgroup analysis of RAR for all-cause and cardiovascular mortality.

In the subgroup analysis, we evaluated the prognostic value of ALI and RAR across different populations. ALI showed no significant interaction in any subgroup (*p* for interaction >0.05), indicating that its prognostic impact on heart failure patients remained stable across different populations. In contrast, RAR exhibited a significant interaction in the sex-stratified analysis (*p* for interaction <0.05). Specifically, among male heart failure patients, RAR had a higher hazard ratio (HR), suggesting a stronger prognostic value in this population (Figure 5).

## Discussion

This study employed multiple analytical approaches to explore the relationships between various inflammation/nutrition indicators and mortality outcomes in heart failure (HF) patients, analyzing cohort data from 1,500 individuals. After false discovery rate (FDR) correction for multiple testing, the vast majority of these markers remained robust independent predictors of mortality, although the associations for NAR and HALP with cardiovascular mortality were not significant. Restricted cubic spline (RCS) analysis revealed linear or nonlinear relationships between these markers and mortality risk. Time-dependent ROC curve analysis indicated that RAR exhibited the highest predictive value for 1-year all-cause and cardiovascular mortality, whereas ALI demonstrated superior predictive performance for 3-year, 5-year, and 10-year mortality. Furthermore, random survival forest (RSF) analysis, validated through a training-validation set split, consistently identified ALI as the top-ranking predictor, underscoring its stable and prominent role in risk stratification. Stratified analysis reinforced the prognostic value of ALI across different populations, suggesting its stable predictive capability and broad clinical applicability for risk stratification in HF patients. Subgroup analysis showed that RAR had a stronger prognostic value in male patients.

The loss of significance for NAR and HALP after FDR correction (*q* > 0.05) for cardiovascular mortality is likely attributable to diminished statistical power following stringent multiple testing adjustment. Associations with more modest effect sizes may not survive this correction, which does not negate their potential biological relevance but indicates that their evidence is less robust than that for stronger predictors like RAR and ALI in our cohort.

MAR and NAR, as emerging composite biomarkers of inflammation and nutritional status, may conceptually be inspired by other ratio-based indices (such as NLR and PLR). The significance of these markers in cancer prognosis (19, 20) has been well established and is gradually being extended to inflammatory diseases (21, 22) and cardiovascular conditions (23, 24). This study is the first to evaluate the prognostic value of MAR and NAR for both all-cause and cardiovascular mortality in patients with HF.

The prognostic utility of PNI (25), RAR (26), HALP score (27), ALI (28), GNRI (29), and CONUT score (30) in HF patients has been discussed in previous studies. Consistent with prior findings, our study confirmed that these markers are independent prognostic indicators for HF patients. However, no previous research has systematically compared multiple inflammation/nutrition markers to evaluate their prognostic roles and relative advantages in HF prognosis. Our findings indicate that RAR was the most effective predictor of 1-year mortality, while ALI exhibited the highest predictive value for 3-year, 5-year, and 10-year mortality, potentially reflecting distinct mechanisms influencing short-term versus long-term prognosis.

A possible explanation for this discrepancy is that RAR integrates red blood cell distribution width (RDW) and albumin levels, primarily reflecting acute inflammatory responses, nutritional status, and hemodynamic changes, which may have a more immediate impact on short-term mortality risk in HF patients (26). RDW represents variability in red blood cell volume and is associated with impaired bone marrow hematopoiesis, heightened inflammatory responses, and reduced oxygen-carrying capacity. Elevated RDW may indicate inflammation activation, iron metabolism disorders, and shortened red blood cell lifespan, all of which can accelerate disease progression and contribute to increased short-term mortality risk in HF patients (31). Previous studies have demonstrated that elevated RDW is closely related to short-term hospitalization rates and mortality in HF and may serve as an important predictor of acute HF decompensation or sudden cardiac death (32, 33). Moreover, decreased albumin levels reflect acute malnutrition and heightened inflammation (34). Albumin is a critical marker of chronic inflammation and nutritional status, and low albumin levels indicate acute disease exacerbation, fluid retention, and hepatic dysfunction, potentially leading to higher short-term mortality risk (35). In acute decompensated HF (e.g., congestion, hypoperfusion, hepatic congestion), serum albumin levels typically decrease (36). Therefore, low albumin levels in the short term may serve as a key predictor of poor prognosis. Given that RAR combines RDW, which reflects erythrocyte variability and systemic inflammation, with albumin, which indicates malnutrition, it may be particularly sensitive for predicting short-term mortality risk, such as 1-year mortality.

In contrast, ALI is composed of BMI, albumin, and the neutrophil-to-lymphocyte ratio (NLR), offering a more comprehensive reflection of chronic inflammation, nutritional status, and immune dysregulation, which could have a more significant impact on the long-term progression of HF. NLR, a key component of ALI, reflects chronic inflammation and immune imbalance, both of which are recognized as important drivers of HF progression (37, 38). HF patients with prolonged survival often experience chronic inflammation, immune dysfunction, and cardiac remodeling—factors that may hold greater predictive value in the long term (39, 40). Additionally, albumin and BMI, core components of ALI, provide long-term insights into a patient’s metabolic reserves, muscle mass, and chronic pathological state. Low BMI and hypoalbuminemia are associated with HF-related cachexia, muscle loss (sarcopenia), and chronic energy expenditure syndromes, all of which are critical determinants of long-term mortality (41–44). Thus, the components of ALI are more indicative of chronic inflammation and nutritional status, making them potentially more stable markers for long-term mortality risk assessment. This explains why ALI exhibited superior predictive performance for 3-year, 5-year, and 10-year mortality, suggesting its suitability for long-term prognosis evaluation.

Inflammation is a well-established contributor to HF progression, with significant impact on disease trajectory and outcomes. Targeted interventions including anti-inflammatory therapies (45) and dietary adjustments (46) have shown promise in reducing cardiovascular events and improving prognosis in HF patients. Similarly, improving nutritional status is a modifiable and clinically important goal (47), given its strong association with mortality. It is noteworthy that nutritional status, rather than body mass index (BMI) alone, is a key modulator of heart failure prognosis. Emerging evidence (48) indicates that the protective effect of obesity in HF patients is confined to those who are well-nourished, while this association vanishes in malnourished individuals. This underscores the limitations of the BMI-based “obesity paradox” for prognostic evaluation, as evidence (49) highlights that nutritional status, rather than BMI alone, is a more central factor in risk stratification. Compared to single markers such as C-reactive protein (CRP) or BMI, composite indices offer a multidimensional approach to risk assessment and allow dynamic monitoring of treatment effects. The prognostic value of RAR and ALI may vary across HF subgroups. In acute decompensated HF, characterized by intense systemic inflammation and rapid nutritional decline, RAR may provide a sensitive and practical tool for short-term risk stratification. Elevated RAR reflects both inflammation and hypoalbuminemia, enabling early identification of high-risk patients who may benefit from timely interventions such as anti-inflammatory therapy and nutritional support. In patients with chronic HF, long-term outcomes are more influenced by low-grade inflammation and progressive malnutrition. In this population, ALI appears to be a stronger predictor of long-term mortality. Persistently low ALI may signal the need for optimized disease management, including adjustments to anti-inflammatory and neurohormonal therapies (such as SGLT2 inhibitors, ARNI), individualized dietary strategies, and the consideration of novel therapies like GLP-1 receptor agonists, which have recently demonstrated efficacy in improving symptoms and physical function in patients with HFpEF (50). Integrating ALI into routine risk assessment for stable HF may help guide personalized, prevention-oriented care.

Future research should further investigate the underlying mechanisms connecting ALI and RAR to HF progression and assess their potential role in guiding individualized therapeutic strategies.

### Strengths and limitations

This study possesses multiple strengths. First, it is among the first to systematically compare and evaluate multiple inflammation/nutrition indicators in assessing the prognosis of heart failure (HF) patients, providing a more comprehensive perspective on their predictive capabilities. By leveraging data from the NHANES database, which includes a diverse population, the study enhances the generalizability of its findings to real-world clinical settings. Additionally, the use of restricted cubic spline (RCS) analysis allowed for a precise examination of the linear and nonlinear relationships between various inflammation/nutrition markers and mortality risk. The integration of time-dependent ROC curves and random survival forest (RSF) analysis further ensured a robust and reliable comparison of predictive performance. Moreover, the study assessed the prognostic utility of these indicators across different time points (1-year, 3-year, 5-year, and 10-year mortality), providing valuable insights into both short-term and long-term risk stratification.

However, this study also has certain limitations. First, the diagnosis of HF in NHANES was based on self-reporting, which may introduce recall bias and prevents differentiation between HFrEF and HFpEF. This limitation in case definition should be considered when interpreting our findings. Second, the ascertainment of causes of death was based on ICD-10 codes from the National Death Index (NDI). This is an inherent limitation of all NHANES-based mortality studies but may involve misclassification bias. While this does not invalidate the robust associations observed with all-cause mortality, it warrants cautious interpretation of the cardiovascular-specific mortality findings. Given its observational design, causal relationships cannot be established, and despite statistical adjustments, residual confounding (such as detailed data on heart failure medication use) remains a potential concern. Additionally, as NHANES is a cross-sectional survey, selection bias may affect the generalizability of the findings. Lastly, while this study underscores the prognostic value of these inflammation/nutrition markers, independent validation in other HF cohorts is necessary to confirm the reproducibility and clinical utility of the findings.

## Conclusion

In summary, ALI demonstrated the strongest predictive value for long-term prognosis and may aid in guiding long-term pharmacologic adjustments and nutritional optimization strategies in patients with stable heart failure. In contrast, RAR showed the highest accuracy in predicting 1-year mortality and may facilitate early identification of high-risk individuals, enabling timely initiation of anti-inflammatory or nutritional interventions. These findings suggest that ALI and RAR hold potential as practical tools for clinical risk stratification at different stages of heart failure. Prospective studies are warranted to validate these results and to further explore whether ALI- and RAR-based interventions can improve outcomes in the heart failure population.

## Data Availability

Publicly available datasets were analyzed in this study. This data can be found here: https://wwwn.cdc.gov/nchs/nhanes/Default.aspx.
