# Supplementary material for: Association between inflammation- and nutrition-related indicators and mortality in patients with heart failure: a cohort study
Source: Front Nutr. 2025 Oct 28;12:1617069. doi: 10.3389/fnut.2025.1617069 (PMC12602239; doi:10.3389/fnut.2025.1617069)
Supplement: Supplementary file 1 [file Table_1.DOCX]

**Table S1**. Details of inflammation/nutrition- based indicators utilized in the study.

| **Indicators** | **Calculation formula** |
| --- | --- |
| NLR | neutrophil count (×10^3^/µL)/ lymphocyte count (×10^3^/µL) |
| ALI | BMI (kg/m2) × albumin(g/dl)/ NLR |
| MAR | monocyte count (×10^3^/µL)/ albumin (g/ L) |
| NAR | neutrophil count (×10^3^/µL)/ albumin (g/ L) |
| RAR | RDW (%)/ albumin (g/ L) |
| PNI | albumin (g/ L) + 5 × Total lymphocyte count (10^3^/µL) |
| GNRI | 1.489×albumin (g/L) + (41.7× current weight/ IBW) |
| HALP score | [hemoglobin (g/ L) × albumin (g/ L) × lymphocytes (×10^3^/µL)]/ platelets (×10^3^/µL) |
| CONUT score | albumin score + Total lymphocyte score + Total cholesterol score. |
| Calculation formula of CONUT score   \| Serum albumin  (g/dL) \| albumin score \| Total lymphocyte  (count/µL) \| Total lymphocyte  score \| Total cholesterol (mg/dL) \| Total cholesterol  score \| \| \| --- \| --- \| --- \| --- \| --- \| --- \| --- \| \| ≥3.5 \| 0 \| ≥1,600 \| 0 \| ≥180 \| \| 0 \| \| 3.0–3.49 \| 2 \| 1,200-1,599 \| 1 \| 140–179 \| \| 1 \| \| 2.5–2.99 \| 4 \| 800–1,199 \| 2 \| 100–139 \| \| 2 \| \| <2.5 \| 6 \| <800 \| 3 \| <100 \| \| 3 \| | |

ALI, Advanced lung cancer inflammation index; MAR, Monocyte- to- albumin ratio; NAR, Neutrophil- to- albumin ratio; RAR, Red cell distribution width- albumin ratio; PNI, Prognostic nutritional index; GNRI, Geriatric nutrition risk index; HALP, Hemoglobin, albumin, lymphocyte, and platelet; CONUT, Controlling nutritional status. RDW, Red blood cell distribution width; IBW, ideal body weight. The ideal body weight was calculated as follows: 0.75 × height (cm)-62.5 for male patients, 0.60 × height (cm)-40 for female patients.

| **Variable** | **Model1 (ALI)** | **Model2 (MAR)** | **Model3 (NAR)** | **Model4 (RAR)** | **Model5 (PNI)** | **Model6 (GNRI)** | **Model7 (HALP)** | **Model8 (CONUT)** |
| --- | --- | --- | --- | --- | --- | --- | --- | --- |
| Indicator | 1.034 | 1.017 | 1.028 | 1.033 | 1.020 | 1.037 | 1.019 | 1.022 |
| Age | 1.257 | 1.247 | 1.249 | 1.246 | 1.248 | 1.272 | 1.247 | 1.248 |
| Gender | 1.082 | 1.082 | 1.081 | 1.088 | 1.082 | 1.091 | 1.094 | 1.089 |
| Race | 1.057 | 1.053 | 1.064 | 1.060 | 1.054 | 1.052 | 1.053 | 1.053 |
| Education | 1.025 | 1.024 | 1.025 | 1.027 | 1.026 | 1.024 | 1.024 | 1.025 |
| Smoke | 1.085 | 1.087 | 1.089 | 1.082 | 1.084 | 1.085 | 1.080 | 1.080 |
| Drink | 1.094 | 1.094 | 1.094 | 1.096 | 1.094 | 1.095 | 1.094 | 1.094 |
| Hypertension | 1.038 | 1.038 | 1.037 | 1.037 | 1.037 | 1.044 | 1.036 | 1.037 |
| Diabetes | 1.279 | 1.281 | 1.284 | 1.283 | 1.281 | 1.298 | 1.279 | 1.284 |
| Coronary artery disease | 1.044 | 1.044 | 1.046 | 1.044 | 1.044 | 1.046 | 1.045 | 1.045 |
| Stroke | 1.033 | 1.013 | 1.013 | 1.013 | 1.013 | 1.014 | 1.013 | 1.014 |
| Cancer | 1.050 | 1.052 | 1.050 | 1.051 | 1.051 | 1.052 | 1.051 | 1.050 |
| HbA1c | 1.274 | 1.272 | 1.270 | 1.270 | 1.266 | 1.269 | 1.268 | 1.269 |
| eGFR | 1.232 | 1.220 | 1.219 | 1.237 | 1.235 | 1.219 | 1.227 | 1.230 |
| ALT | 1.510 | 1.511 | 1.512 | 1.517 | 1.510 | 1.512 | 1.510 | 1.510 |
| AST | 1.496 | 1.496 | 1.496 | 1.500 | 1.498 | 1.498 | 1.496 | 1.500 |

**Table S2.** Generalized Variance Inflation Factor (GVIF) Values for Covariates in the Fully Adjusted Cox Regression Models.

**Note:** GVIF^(1/(2*Df)) values are presented. A value below 5 indicates no severe multicollinearity. Df, degrees of freedom. ALI, Advanced lung cancer inflammation index; MAR, Monocyte- to- albumin ratio; NAR, Neutrophil- to- albumin ratio; RAR, Red cell distribution width- albumin ratio; PNI, Prognostic nutritional index; GNRI, Geriatric nutrition risk index; HALP, Hemoglobin, albumin, lymphocyte, and platelet; CONUT, Controlling nutritional status; Indicator, the inflammation/nutrition- based indicator unique to each model; HbA1c, Glycosylated hemoglobin, type A1C; eGFR, estimated glomerular filtration rate; AST, Aspartate aminotransferase; ALT, Alanine aminotransferase.

**Table S3.** Time-dependent C-index of inflammation/nutrition- based indicators for predicting all-cause mortality in heart failure patients.

|  | **C-index** | | | |
| --- | --- | --- | --- | --- |
|  | **1‐year** | **3‐years** | **5-years** | **10-years** |
| ALI | 0.672 (0.611, 0.734) | **0.671 (0.638, 0.704)** | **0.662 (0.635, 0.689)** | **0.653 (0.631, 0.676)** |
| MAR | 0.593 (0.535, 0.651) | 0.611 (0.577, 0.645) | 0.596 (0.568, 0.624) | 0.593 (0.570, 0.616) |
| NAR | 0.589 (0.531, 0.646) | 0.606 (0.573, 0.639) | 0.589 (0.561, 0.616) | 0.588 (0.566, 0.611) |
| RAR | **0.752 (0.705, 0.800)** | 0.663 (0.631, 0.696) | 0.639 (0.612, 0.667) | 0.628 (0.605, 0.651) |
| PNI | 0.699 (0.635, 0.763) | 0.653 (0.618, 0.688) | 0.634 (0.605, 0.664) | 0.621 (0.597, 0.645) |
| GNRI | 0.626 (0.561, 0.691) | 0.611 (0.577, 0.646) | 0.607 (0.579, 0.636) | 0.597 (0.573, 0.620) |
| HALP score | 0.364 (0.301, 0.428) | 0.603 (0.568, 0.637) | 0.589 (0.561, 0.618) | 0.582 (0.558, 0.606) |
| CONUT score | 0.655 (0.582, 0.728) | 0.638 (0.596, 0.679) | 0.637 (0.603, 0.671) | 0.623 (0.594, 0.652) |

AUC, Area under the curve; 95% CI, 95% Confidence interval; ALI, Advanced lung cancer inflammation index; MAR, Monocyte- to- albumin ratio; NAR, Neutrophil- to- albumin ratio; RAR, Red cell distribution width- albumin ratio; PNI, Prognostic nutritional index; GNRI, Geriatric nutrition risk index; HALP, Hemoglobin, albumin, lymphocyte, and platelet; CONUT, Controlling nutritional status.

**Table S4.** Time-dependent C-index of inflammation/nutrition- based indicators for predicting CVD mortality in heart failure patients.

|  | **C-index** | | | |
| --- | --- | --- | --- | --- |
|  | **1‐year** | **3‐years** | **5-years** | **10-years** |
| ALI | 0.686 (0.601, 0.771) | **0.686 (0.636, 0.736)** | **0.669 (0.628, 0.709)** | **0.662 (0.627, 0.697)** |
| MAR | 0.607 (0.536, 0.678) | 0.615 (0.567, 0.664) | 0.602 (0.562, 0.642) | 0.609 (0.575, 0.644) |
| NAR | 0.601 (0.530, 0.673) | 0.589 (0.541, 0.637) | 0.569 (0.529, 0.608) | 0.579 (0.544, 0.613) |
| RAR | **0.768 (0.707, 0.829)** | 0.672 (0.623, 0.722) | 0.638 (0.597, 0.680) | 0.632 (0.596, 0.668) |
| PNI | 0.719 (0.635, 0.804) | 0.673 (0.618, 0.728) | 0.648 (0.603, 0.692) | 0.634 (0.595, 0.673) |
| GNRI | 0.610 (0.523, 0.696) | 0.635 (0.584, 0.686) | 0.622 (0.581, 0.664) | 0.613 (0.576, 0.649) |
| HALP score | 0.669 (0.581, 0.757) | 0.624 (0.570, 0.678) | 0.601 (0.557, 0.645) | 0.591 (0.552, 0.629) |
| CONUT score | 0.695 (0.598, 0.792) | 0.668 (0.606, 0.729) | 0.668 (0.618, 0.718) | 0.654 (0.610, 0.698) |

AUC, Area under the curve; 95% CI, 95% Confidence interval; ALI, Advanced lung cancer inflammation index; MAR, Monocyte- to- albumin ratio; NAR, Neutrophil- to- albumin ratio; RAR, Red cell distribution width- albumin ratio; PNI, Prognostic nutritional index; GNRI, Geriatric nutrition risk index; HALP, Hemoglobin, albumin, lymphocyte, and platelet; CONUT, Controlling nutritional status.

**Table S5.** The AUC of inflammation/nutrition-based indicators for predicting mortality in heart failure stratified by sex.

| **Indicators** | **All-cause mortality** | | **CVD mortality** | |
| --- | --- | --- | --- | --- |
|  | AUC (95%CI) | | AUC (95%CI) | |
|  | Male | Female | Male | Female |
| ALI | **0.649 (0.612, 0.686)** | **0.674 (0.633, 0.714)** | **0.621 (0.575, 0.666)** | **0.625 (0.571, 0.678)** |
| MAR | 0.583 (0.545, 0.622) | 0.542 (0.498, 0.586) | 0.580 (0.533, 0.627) | 0.536 (0.480, 0.592) |
| NAR | 0.590 (0.552, 0.629) | 0.562 (0.518, 0.606) | 0.563 (0.517, 0.609) | 0.527 (0.471, 0.584) |
| RAR | 0.575 (0.537, 0.614) | 0.505 (0.461, 0.549) | 0.555 (0.506, 0.604) | 0.508 (0.453, 0.563) |
| PNI | 0.603 (0.565, 0.641) | 0.563 (0.519, 0.607) | 0.594 (0.544, 0.644) | 0.538 (0.482, 0.482) |
| GNRI | 0.602 (0.564, 0.640) | 0.612 (0.569, 0.655) | 0.584 (0.537, 0.631) | 0.589 (0.536, 0.642) |
| HALP score | 0.596 (0.557, 0.634) | 0.592 (0.548, 0.635) | 0.563 (0.514, 0.612) | 0.583 (0.528, 0.637) |
| CONUT score | 0.563 (0.525, 0.602) | 0.537 (0.493, 0.581) | 0.575 (0526, 0.623) | 0.541 (0.485, 0.598) |

AUC, Area under curve; CVD, Cardiovascular disease; CI, Confidence interval; ALI, Advanced lung cancer inflammation index; MAR, Monocyte- to- albumin ratio; NAR, Neutrophil- to- albumin ratio; RAR, Red cell distribution width- albumin ratio; PNI, Prognostic nutritional index; GNRI, Geriatric nutrition risk index; HALP, Hemoglobin, albumin, lymphocyte, and platelet; CONUT, Controlling nutritional status.

#### Table S6. The AUC of inflammation/nutrition-based indicators for predicting mortality in heart failure stratified by age.

| **Indicators** | **All-cause mortality** | | **CVD mortality** | |
| --- | --- | --- | --- | --- |
|  | AUC (95%CI) | | AUC (95%CI) | |
|  | Age≥65 | Age<65 | Age≥65 | Age<65 |
| ALI | **0.645 (0.609, 0.681)** | 0.592 (0.539, 0.646) | **0.588 (0.548, 0.628)** | **0.616 (0.535, 0.697)** |
| MAR | 0.531 (0.495, 0.568) | 0.596 (0.544, 0.648) | 0.534 (0.492, 0.576) | 0.609 (0.533, 0.685) |
| NAR | 0.560 (0.523, 0.597) | **0.600 (0.547, 0.653)** | 0.520 (0.478, 0.561) | 0.615 (0.538, 0.692) |
| RAR | 0.514 (0.477, 0.551) | 0.573 (0.522, 0.624) | 0.518 (0.476, 0.561) | 0.570 (0.491, 0.649) |
| PNI | 0.572 (0.535, 0.608) | 0.556 (0.502, 0.610) | 0.551 (0.508, 0.594) | 0.571 (0.489, 0.654) |
| GNRI | 0.613 (0.576, 0.649) | 0.529 (0.476, 0.583) | 0.578 (0.537, 0.618) | 0.537 (0.461, 0.614) |
| HALP score | 0.606 (0.570, 0.642) | 0.523 (0.470, 0.576) | 0.564 (0.521, 0.606) | 0.534 (0.454, 0.614) |
| CONUT score | 0.517 (0.480, 0.554) | 0.549 (0.496, 0.601) | 0.538 (0.496, 0.581) | 0.556 (0.475, 0.637) |

AUC, Area under curve; CVD, Cardiovascular disease; CI, Confidence interval; ALI, Advanced lung cancer inflammation index; MAR, Monocyte- to- albumin ratio; NAR, Neutrophil- to- albumin ratio; RAR, Red cell distribution width- albumin ratio; PNI, Prognostic nutritional index; GNRI, Geriatric nutrition risk index; HALP, Hemoglobin, albumin, lymphocyte, and platelet; CONUT, Controlling nutritional status.

#### Table S7. The AUC of inflammation/nutrition-based indicators for predicting mortality in heart failure stratified by BMI.

| **Indicators** | **All-cause mortality** | | **CVD mortality** | |
| --- | --- | --- | --- | --- |
|  | AUC (95%CI) | | AUC (95%CI) | |
|  | BMI≥25 | BMI<25 | BMI≥25 | BMI<25 |
| ALI | **0.655 (0.625, 0.686)** | **0.633 (0.567, 0.699)** | **0.623 (0.584, 0.662)** | **0.609 (0.533, 0.684)** |
| MAR | 0.560 (0.528, 0.592) | 0.609 (0.542, 0.675) | 0.568 (0.527, 0.609) | 0.546 (0.469, 0.623) |
| NAR | 0.575 (0.543, 0.608) | 0.615 (0.548, 0.682) | 0.544 (0.503, 0.584) | 0.569 (0.492, 0.584) |
| RAR | 0.537 (0.505, 0.570) | 0.560 (0.492, 0.628) | 0.532 (0.490, 0.574) | 0.554 (0.478, 0.630) |
| PNI | 0.587 (0.555, 0.619) | 0.574 (0.507, 0.641) | 0.568 (0.525, 0.610) | 0.574 (0.495, 0.653) |
| GNRI | 0.599 (0.567, 0.631) | 0.531 (0.463, 0.599) | 0.591 (0.551, 0.631) | 0.561 (0.482, 0.640) |
| HALP score | 0.596 (0.564, 0.628) | 0.548 (0.481, 0.616) | 0.578 (0.536, 0.619) | 0.528 (0.447, 0.610) |
| CONUT score | 0.560 (0.528, 0.592) | 0.523 (0.455, 0.592) | 0.564 (0.522, 0.606) | 0.532 (0.455, 0.608) |

AUC, Area under curve; CVD, Cardiovascular disease; CI, Confidence interval; ALI, Advanced lung cancer inflammation index; MAR, Monocyte- to- albumin ratio; NAR, Neutrophil- to- albumin ratio; RAR, Red cell distribution width- albumin ratio; PNI, Prognostic nutritional index; GNRI, Geriatric nutrition risk index; HALP, Hemoglobin, albumin, lymphocyte, and platelet; CONUT, Controlling nutritional status.

#### Table S8. The AUC of inflammation/nutrition-based indicators for predicting mortality in heart failure stratified by hypertension status.

| Indicators | **All-cause mortality** | | **CVD mortality** | |
| --- | --- | --- | --- | --- |
|  | AUC (95%CI) | | AUC (95%CI) | |
|  | No Hypertension | Hypertension | No Hypertension | Hypertension |
| ALI | **0.636 (0.569, 0.702)** | **0.665 (0.635, 0.695)** | 0.601 (0.520, 0.682) | **0.628 (0.590, 0.667)** |
| MAR | 0.547 (0.478, 0.616) | 0.574 (0.542, 0.606) | **0.608 (0.525, 0.691)** | 0.552 (0.512, 0.592) |
| NAR | 0.586 (0.517, 0.654) | 0.578 (0.547, 0.610) | 0.590 (0.508, 0.673) | 0.538 (0.499, 0.578) |
| RAR | 0.559 (0.490, 0.628) | 0.537 (0.505, 0.569) | 0.593 (0.511, 0.675) | 0.523 (0.482, 0.564) |
| PNI | 0.562 (0.493, 0.630) | 0.591 (0.560, 0.623) | 0.515 (0.430, 0.600) | 0.584 (0.543, 0.626) |
| GNRI | 0.517 (0.448, 0.586) | 0.631 (0.600, 0.662) | 0.505 (0.426, 0.585) | 0.601 (0.562, 0.641) |
| HALP score | 0.548 (0.479, 0.616) | 0.601 (0.569, 0.632) | 0.513 (0.428, 0.598) | 0.582 (0.542, 0.623) |
| CONUT score | 0.556 (0.488, 0.625) | 0.553 (0.521, 0.585) | 0.549 (0.466, 0.632) | 0.564 (0.523, 0.605) |

AUC, Area under curve; CVD, Cardiovascular disease; CI, Confidence interval; ALI, Advanced lung cancer inflammation index; MAR, Monocyte- to- albumin ratio; NAR, Neutrophil- to- albumin ratio; RAR, Red cell distribution width- albumin ratio; PNI, Prognostic nutritional index; GNRI, Geriatric nutrition risk index; HALP, Hemoglobin, albumin, lymphocyte, and platelet; CONUT, Controlling nutritional status.

#### Table S9. The AUC of inflammation/nutrition-based indicators for predicting mortality in heart failure stratified by diabetes status.

| Indicators | **All-cause mortality** | | **CVD mortality** | |
| --- | --- | --- | --- | --- |
|  | AUC (95%CI) | | AUC (95%CI) | |
|  | No diabetes | diabetes | No diabetes | diabetes |
| ALI | **0.659 (0.622, 0.697)** | **0.663 (0.622, 0.703)** | **0.639 (0.592, 0.686)** | **0.606 (0.555, 0.657)** |
| MAR | 0.589 (0.551, 0.628) | 0.537 (0.494, 0.580) | 0.568 (0.519, 0.617) | 0.555 (0.501, 0.609) |
| NAR | 0.578 (0.539, 0.617) | 0.575 (0.532, 0.617) | 0.558 (0.509, 0.607) | 0.533 (0.481, 0.585) |
| RAR | 0.546 (0.506, 0.585) | 0.521 (0.478, 0.564 | 0.527 (0.476, 0.578) | 0.537 (0.484, 0.589) |
| PNI | 0.581 (0.542, 0.620) | 0.586 (0.544, 0.628) | 0.560 (0.509, 0.611) | 0.580 (0.525, 0.635) |
| GNRI | 0.622 (0.583, 0.660) | 0.606 (0.564, 0.564) | 0.594 (0.547, 0.641) | 0.584 (0.533, 0.636) |
| HALP score | 0.580 (0.541, 0.619) | 0.602 (0.560, 0.644) | 0.554 (0.503, 0.605) | 0.584 (0.531, 0.637) |
| CONUT score | 0.562 (0.523, 0.602) | 0.537 (0.494, 0.580) | 0.568 (0.518, 0.617) | 0.553 (0.498, 0.607) |

AUC, Area under curve; CVD, Cardiovascular disease; CI, confidence interval; ALI, Advanced lung cancer inflammation index; MAR, Monocyte- to- albumin ratio; NAR, Neutrophil- to- albumin ratio; RAR, Red cell distribution width- albumin ratio; PNI, Prognostic nutritional index; GNRI, Geriatric nutrition risk index; HALP, Hemoglobin, albumin, lymphocyte, and platelet; CONUT, Controlling nutritional status.

#### Table S10. Baseline characteristics of patients with heart failure based on ALI quantile in NHANES 1999–2018.

| **Variables** | **ALI quantile** | | | | |
| --- | --- | --- | --- | --- | --- |
|  | Quantile1  (n = 375) | Quantile2  (n = 375) | Quantile3  (n = 375) | Quantile4  (n = 375) | *P* |
| Age | 76.0 (67.0, 80.0) | 72.0 (63.0, 79.5) | 68.0 (59.0, 77.0) | 63.0 (55.0, 72.0) | <0.001 |
| Gender, n (%) |  |  |  |  | 0.021 |
| female | 146 (38.93) | 153 (40.80) | 177 (47.20) | 181 (48.27) |  |
| male | 229 (61.07) | 222 (59.20) | 198 (52.80) | 194 (51.73) |  |
| Race, n (%) |  |  |  |  | <0.001 |
| Mexican American | 37 (9.87) | 40 (10.67) | 49 (13.07) | 36 (9.60) |  |
| Non- Hispanic White | 249 (66.40) | 231 (61.60) | 193 (51.47) | 147 (39.20) |  |
| Non- Hispanic Black | 55 (14.67) | 65 (17.33) | 79 (21.07) | 144 (38.40) |  |
| Others | 34 (9.07) | 39 (10.40) | 54 (14.40) | 48 (12.80) |  |
| Education level, n (%) |  |  |  |  | 0.498 |
| Below high school | 152 (40.53) | 142 (38.07) | 146 (38.93) | 151 (40.37) |  |
| High school | 101 (26.93) | 98 (26.27) | 83 (22.13) | 85 (22.73) |  |
| Above high school | 122 (32.53) | 133 (35.66) | 146 (38.93) | 138 (36.90) |  |
| Smoke, n (%) |  |  |  |  | 0.003 |
| No | 129 (34.40) | 129 (34.40) | 171 (45.60) | 154 (41.18) |  |
| Yes | 246 (65.60) | 246 (65.60) | 204 (54.40) | 220 (58.82) |  |
| Drink, n (%) |  |  |  |  | 0.443 |
| No | 56 (16.09) | 48 (13.48) | 60 (17.34) | 50 (13.97) |  |
| Yes | 292 (83.91) | 308 (86.52) | 286 (82.66) | 308 (86.03) |  |
| Hypertension, n (%) |  |  |  |  | 0.406 |
| No | 72 (19.20) | 72 (19.20) | 71 (18.93) | 57 (15.20) |  |
| Yes | 303 (80.80) | 303 (80.80) | 304 (81.07) | 318 (84.80) |  |
| Diabetes, n (%) |  |  |  |  | 0.538 |
| No | 214 (57.07) | 199 (53.07) | 195 (52.00) | 204 (54.40) |  |
| Yes | 161 (42.93) | 176 (46.93) | 180 (48.00) | 171 (45.60) |  |
| Coronary artery disease, n (%) |  |  |  |  | 0.162 |
| No | 208 (58.26) | 203 (55.77) | 202 (55.80) | 231 (62.94) |  |
| Yes | 149 (41.74) | 161 (44.23) | 160 (44.20) | 136 (37.06) |  |
| Stroke, n (%) |  |  |  |  | 0.299 |
| No | 297 (79.41) | 290 (77.33) | 297 (79.62) | 310 (82.89) |  |
| Yes | 77 (20.59) | 85 (22.67) | 76 (20.38) | 64 (17.11) |  |
| Cancer, n (%) |  |  |  |  | 0.002 |
| No | 278 (74.13) | 286 (76.27) | 309 (83.06) | 311 (83.38) |  |
| Yes | 97 (25.87) | 89 (23.73) | 63 (16.94) | 62 (16.62) |  |
| HbA1c, % | 5.80 (5.40, 6.40) | 5.90 (5.50, 6.50) | 6.00 (5.50, 6.95) | 6.00 (5.50, 6.70) | 0.001 |
| Creatinine, μmol/L | 1.20 (0.94, 1.60) | 1.07 (0.85, 1.38) | 1.00 (0.82, 1.25) | 0.99 (0.80, 1.20) | <0.001 |
| eGFR, mL/min/1.73m^2 | 55.41 (38.86, 74.49) | 63.96 (47.51, 84.26) | 69.95 (52.53, 89.31) | 77.55 (87.69, 94.43) | <0.001 |
| ALT, mmol/L | 18.00 (14.00, 23.00) | 18.00 (14.00, 24.00) | 19.00 (15.00, 26.00) | 21.00 (17.00, 29.00) | <0.001 |
| AST, mmol/L | 23.00 (19.00, 27.00) | 22.00 (19.00, 27.00) | 23.00 (19.00, 27.00) | 23.00 (19.00, 28.00) | 0.333 |
| **Inflammation and nutritional indicators** | | | | | |
| Lymphocyte, 10^3^/µL | 1.20 (1.00, 1.50) | 1.70 (1.40, 2.10) | 2.10 (1.70, 2.50) | 2.50 (2.00, 3.00) | <0.001 |
| Monocyte, 10^3^/µL | 0.60 (0.50, 0.70) | 0.60 (0.50, 0.75) | 0.60 (0.50, 0.70) | 0.60 (0.50, 0.70) | 0.060 |
| Neutrophil, 10^3^/µL | 5.60 (4.50, 6.85) | 4.70 (3.70, 5.80) | 4.20 (3.45, 5.20) | 3.40 (2.60, 4.30) | <0.001 |
| Hemoglobin, g/dL | 13.40 (12.10, 14.50) | 13.50 (12.50, 14.80) | 13.80 (12.70, 14.80) | 13.80 (12.70, 14.70) | <0.001 |
| RDW, % | 14.10 (13.20, 15.30) | 13.70 (13.00, 14.80) | 13.50 (12.90, 14.50) | 13.60 (12.85, 14.50) | <0.001 |
| Platelet, 10^3^/µL | 213.00 (176.50, 266.00) | 217.00 (178.00, 267.50) | 225.00 (184.50, 274.00) | 219.00 (180.50, 259.00) | 0.339 |
| BMI, kg/m2 | 26.97 (23.58, 30.44) | 29.00 (25.59, 33.84) | 31.31 (27.20, 36.61) | 34.24 (29.24, 41.02) | <0.001 |
| Total cholesterol, mmol/L | 4.32 (3.57, 5.22) | 4.50 (3.69, 5.32) | 4.63 (3.83, 5.54) | 4.76 (3.98, 5.52) | <0.001 |
| Serum albumin, g/L | 40.00 (37.00, 42.00) | 41.00 (38.50, 44.00) | 41.00 (39.00, 43.00) | 41.00 (39.00, 43.00) | <0.001 |

ALI, Advanced lung cancer inflammation index; HbA1c, Glycosylated hemoglobin, type A1C; eGFR, estimated glomerular filtration rate; AST, Aspartate aminotransferase; ALT, Alanine aminotransferase; RDW, Red cell distribution width; BMI, Body mass index; TC, Total cholesterol.

#### Table S11. Baseline characteristics of patients with heart failure based on RAR quantile in NHANES 1999–2018.

| **Variables** | **RAR quantile** | | | | |
| --- | --- | --- | --- | --- | --- |
|  | Quantile 1  (n = 375) | Quantile 2  (n = 372) | Quantile 3  (n = 378) | Quantile 4  (n = 375) | *P* |
| Age | 69 (59, 77) | 70 (60, 80) | 71 (61, 80) | 70 (61, 79) | 0.170 |
| Gender, n (%) |  |  |  |  | <0.001 |
| female | 126 (33.60) | 172 (46.24) | 169 (44.71) | 190 (50.67) |  |
| male | 249 (66.40) | 200 (53.76) | 209 (55.29) | 185 (49.33) |  |
| Race, n (%) |  |  |  |  | <0.001 |
| Mexican American | 45 (12.00) | 50 (13.44) | 33 (8.73) | 34 (9.07) |  |
| Non- Hispanic White | 237 (63.20) | 222 (59.68) | 187 (49.47) | 174 (46.40) |  |
| Non- Hispanic Black | 44 (11.73) | 57 (15.32) | 110 (29.10) | 132 (35.20) |  |
| Others | 49 (13.07) | 43 (11.56) | 48 (12.70) | 35 (9.33) |  |
| Education level, n (%) |  |  |  |  | 0.043 |
| Below high school | 161 (42.93) | 152 (40.97) | 135 (35.90) | 143 (38.13) |  |
| High school | 74 (19.73) | 93 (25.07) | 113 (30.05) | 87 (23.20) |  |
| Above high school | 140 (37.33) | 126 (33.96) | 128 (34.04) | 145 (38.67) |  |
| Smoke, n (%) |  |  |  |  | 0.576 |
| No | 146 (38.93) | 149 (40.05) | 153 (40.58) | 135 (36.00) |  |
| Yes | 229 (61.07) | 223 (59.95) | 224 (59.42) | 240 (64.00) |  |
| Drink, n (%) |  |  |  |  | 0.322 |
| No | 50 (13.97) | 63 (17.90) | 47 (13.28) | 54 (15.70) |  |
| Yes | 308 (86.03) | 289 (82.10) | 307 (86.72) | 290 (84.30) |  |
| Hypertension, n (%) |  |  |  |  | 0.008 |
| No | 87 (23.20) | 66 (17.74) | 68 (17.99) | 51 (13.60) |  |
| Yes | 288 (76.80) | 306 (82.26) | 310 (82.01) | 324 (86.40) |  |
| Diabetes, n (%) |  |  |  |  | <0.001 |
| No | 249 (66.40) | 220 (59.14) | 195 (51.59) | 148 (39.47) |  |
| Yes | 126 (33.60) | 152 (40.86) | 183 (48.41) | 227 (60.53) |  |
| Coronary artery disease, n (%) |  |  |  |  | 0.249 |
| No | 209 (57.42) | 195 (54.47) | 216 (59.18) | 224 (61.71) |  |
| Yes | 155 (42.58) | 163 (45.53) | 149 (40.82) | 139 (38.29) |  |
| Stroke, n (%) |  |  |  |  | 0.562 |
| No | 306 (81.60) | 292 (78.71) | 305 (80.90) | 291 (78.02) |  |
| Yes | 69 (18.40) | 79 (21.29) | 72 (19.10) | 82 (21.98) |  |
| Cancer, n (%) |  |  |  |  | 0.570 |
| No | 299 (79.95) | 297 (80.05) | 303 (80.16) | 285 (76.61) |  |
| Yes | 75 (20.05) | 74 (19.95) | 75 (19.84) | 87 (23.39) |  |
| HbA1c, % | 5.70 (5.30, 6.20) | 5.80 (5.50, 6.40) | 6.00 (5.60, 6.80) | 6.20 (5.60, 7.00) | <0.001 |
| Creatinine, μmol/L | 1.00 (0.80, 1.20) | 1.01 (0.84, 1.25) | 1.08 (0.85, 1.40) | 1.20 (0.92, 1.60) | <0.001 |
| eGFR, mL/min/1.73m^2 | 76.05 (57.93, 91.03) | 67.37 (50.46, 87.86) | 65.28 (44.15, 83.42) | 57.66 (37.69, 78.87) | <0.001 |
| ALT, mmol/L | 21.00 (17.00, 27.00) | 20.00 (15.00, 26.00) | 19.00 (15.00, 24.00) | 17.00 (14.00, 22.00) | <0.001 |
| AST, mmol/L | 24.00 (20.00, 28.00) | 23.00 (20.00, 28.00) | 22.00 (19.00, 27.00) | 21.00 (18.00, 27.00) | <0.001 |
| **Inflammation and nutritional indicators** | | | | | |
| Lymphocyte, 10^3^/µL | 1.90 (1.50, 2.50) | 1.90 (1.50, 2.50) | 1.80 (1.30, 2.30) | 1.70 (1.30, 2.20) | <0.001 |
| Monocyte, 10^3^/µL | 0.60 (0.50, 0.70) | 0.60 (0.50, 0.70) | 0.60 (0.50, 0.80) | 0.60 (0.50, 0.80) | 0.001 |
| Neutrophil, 10^3^/µL | 4.40 (3.35, 5.30) | 4.40 (3.50, 5.60) | 4.45 (3.50, 5.70) | 4.60 (3.40, 5.85) | 0.171 |
| Hemoglobin, g/dL | 14.50 (13.55, 15.50) | 13.90 (13.10, 14.90) | 13.45 (12.40, 14.40) | 12.50 (11.40, 13.60) | <0.001 |
| RDW, % | 12.70 (12.30, 13.10) | 13.40 (12.90, 13.83) | 14.10 (13.50, 14.70) | 15.60 (14.90, 16.85) | <0.001 |
| Platelet, 10^3^/µL | 221.00 (181.50, 259.00) | 219.00 (182.75, 267.00) | 213.00 (176.25, 266.75) | 220.00 (177.00, 277.00) | 0.627 |
| BMI, kg/m2 | 28.47 (25.49, 32.70) | 29.40 (26.03, 34.45) | 31.40 (26.50, 36.52) | 31.90 (26.44, 39.48) | <0.001 |
| Total cholesterol, mmol/L | 4.78 (4.11, 5.59) | 4.73 (3.87, 5.57) | 4.45 (3.75, 5.30) | 4.27 (3.50, 5.05) | <0.001 |
| Serum albumin, g/L | 44.00 (42.00, 46.00) | 41.00 (40.00, 43.00) | 40.00 (38.00, 41.00) | 37.00 (35.00, 39.00) | <0.001 |

RAR, Red cell distribution width- albumin ratio; HbA1c, Glycosylated hemoglobin, type A1C; eGFR, estimated glomerular filtration rate; AST, Aspartate aminotransferase; ALT, Alanine aminotransferase; RDW, Red cell distribution width; BMI, Body mass index; TC, Total cholesterol.


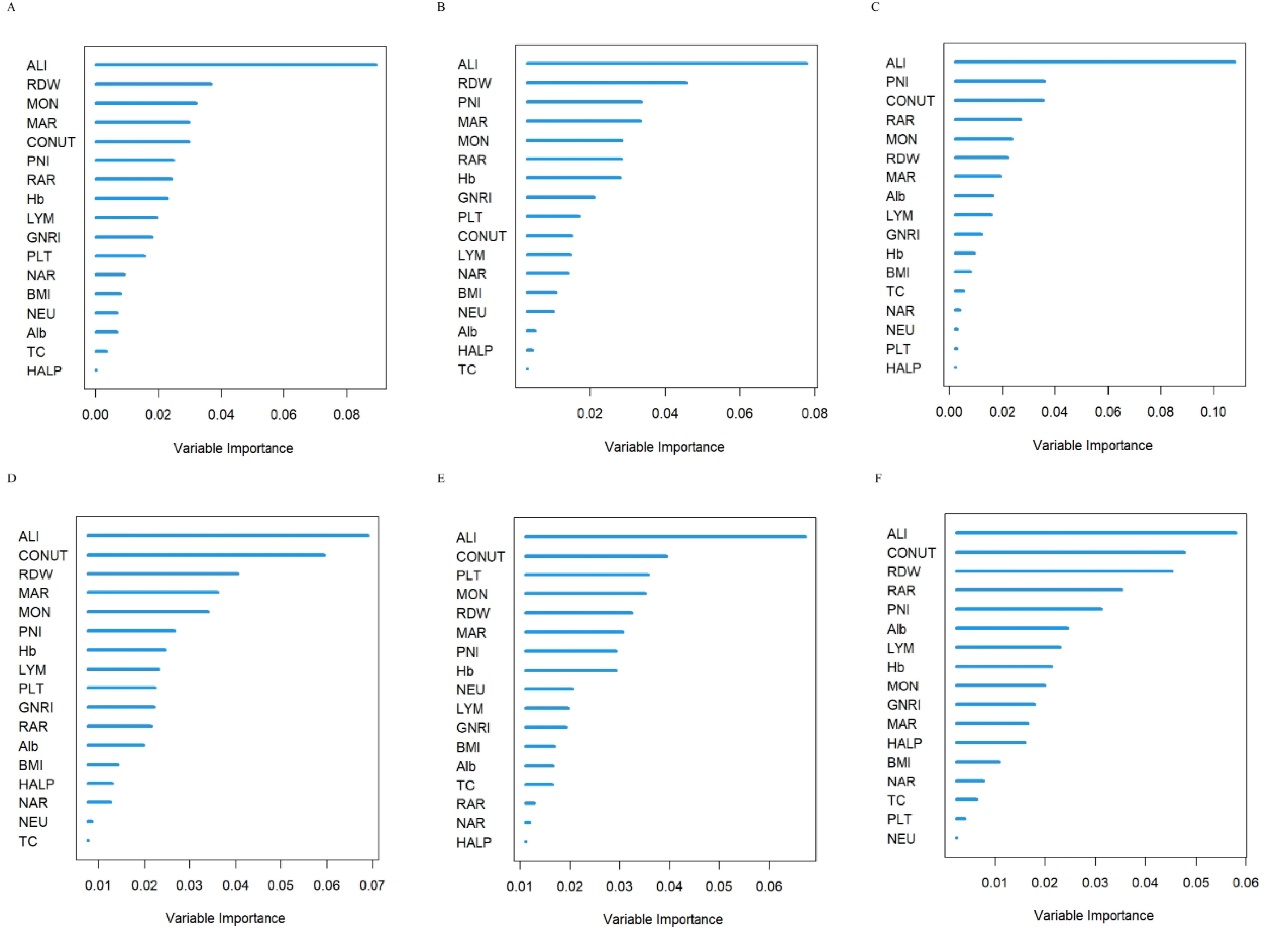


Figure S1.

1. Variable Importance from Random Survival Forest for All-Cause Mortality (Total Cohort)
2. Variable Importance from Random Survival Forest for All-Cause Mortality (Training Set)
3. Variable Importance from Random Survival Forest for All-Cause Mortality (Internal Validation Set)
4. Variable Importance from Random Survival Forest for Cardiovascular Mortality (Total Cohort)
5. Variable Importance from Random Survival Forest for Cardiovascular Mortality (Training Set)
6. Variable Importance from Random Survival Forest for Cardiovascular Mortality (Internal Validation Set)
